# Extension of human GCSF serum half-life by the fusion of albumin binding domain

**DOI:** 10.1038/s41598-021-04560-6

**Published:** 2022-01-13

**Authors:** Fatemeh Yadavar Nikravesh, Samira Shirkhani, Elham Bayat, Yeganeh Talebkhan, Esmat Mirabzadeh, Masoumeh Sabzalinejad, Hooman Aghamirza Moghim Aliabadi, Leila Nematollahi, Yalda Hosseinzadeh Ardakani, Soroush Sardari

**Affiliations:** 1 Biotechnology Research Center, Pasteur Institute of Iran, Tehran, Iran; 2 Department of Molecular Medicine, Pasteur Institute of Iran, Tehran, Iran; 3 Biopharmaceutics and Pharmacokinetic Division, Department of Pharmaceutics, Faculty of Pharmacy, Tehran, Iran

**Keywords:** Biologics, Expression systems, Protein delivery

## Abstract

Granulocyte colony stimulating factor (GCSF) can decrease mortality of patients undergo chemotherapy through increasing neutrophil counts. Many strategies have been developed to improve its blood circulating time. Albumin binding domain (ABD) was genetically fused to N-terminal end of GCSF encoding sequence and expressed as cytoplasmic inclusion bodies within *Escherichia coli*. Biological activity of ABD-GCSF protein was assessed by proliferation assay on NFS-60 cells. Physicochemical properties were analyzed through size exclusion chromatography, circular dichroism, intrinsic fluorescence spectroscopy and dynamic light scattering. Pharmacodynamics and pharmacokinetic properties were also investigated in a neutropenic rat model. CD and IFS spectra revealed that ABD fusion to GCSF did not significantly affect the secondary and tertiary structures of the molecule. DLS and SEC results indicated the absence of aggregation formation. EC50 value of the ABD-GCSF in proliferation of NFS-60 cells was 75.76 pg/ml after 72 h in comparison with control GCSF molecules (Filgrastim: 73.1 pg/ml and PEG-Filgrastim: 44.6 pg/ml). Animal studies of ABD-GCSF represented improved serum half-life (9.3 ± 0.7 h) and consequently reduced renal clearance (16.1 ± 1.4 ml/h.kg) in comparison with Filgrastim (1.7 ± 0.1 h). Enhanced neutrophils count following administration of ABD-GCSF was comparable with Filgrastim and weaker than PEG-Filgrastim treated rats. In vitro and in vivo results suggested the ABD fusion as a potential approach for improving GCSF properties.

## Introduction

Inflammatory cytokines such as tumor necrosis factor alpha (TNF-α), and interleukin 1 beta (IL-1B) are being released early during the course of microbial infections. These factors stimulate NF-κB and C/EBPβ pathways which enhance granulocyte colony-stimulating factor (GCSF) expression^1^ and consequently higher production of granulocytes and stem cells from bone marrow. This hematopoietic cytokine is produced by endothelial and mesothelial cells, macrophages, fibroblasts and monocytes^2^ and is usually administeredto neutropenic individuals after chemotherapy^3^. This growth factor consists of four antiparallel α-helices compromising 174 amino acids (19.6 kDa)^4,5^ with two disulfide bonds (Cys36-Cys42 and Cys64-Cys74), one free cysteine (Cys17) and one O-linked carbohydrate chain attached to Thr133. It has been proved that the carbohydrate moiety has little or no impact on 3D structure and biological activity of GCSF^6^ but the disulfide bonds are necessary for the stability and biological activity of molecule^7,8^. Due to the small size of the molecule, it is usually destroyed with serum proteases and eliminated by the kidney infiltration. Because of its high clearance rate (0.5 to 0.7 ml/min/kg), patients should receive daily which cause patients dissatisfaction and increase treatment costs^9^. Therefore, several strategies have been developed to improve its serum-half life and pharmacokinetic properties. One approach is the attachment of polyethylene glycol (PEG) to the molecule. Although, PEGylation has low immunogenicity, high stability, no electrical charge and high flexibility, it shows some disadvantages including metabolic accumulation within the kidneys and high cost of purification^10,11^. Other suggested approaches are genetically fusion of the target molecule to Fc domain of IgG1^12^, human serum albumin (HASylation)^13^, albumin-binding domain^14–16^, or addition of PAS motif (PASylation)^17^, lipids (Acylation)^18^, XTENylation^11^.

Albumin is the most abundant protein of the plasma^19^ and its serum half-life increases by its attachment to the FcRn^20,21^. It has been well documented that albumin-binding domains (ABDs) can non-covalently bind to the serum albumin^22,23^ and increase the durability of the genetically attached molecule within blood circulation^24–26^. Different types of albumin binding domains are being expressed on the surface of several bacterial families including Staphylococci, and Streptococci^27^. *Finegoldia magna*, an anaerobic gram-positive coccoid bacterium, also expresses ABD (G148-ABD)^28^ which was manipulated to ADB035, a highly stable but highly immunogenic molecule^27^. Further studies developed ABD molecules which were safer and had desirable affinity towards albumin (ABD088, ABD094 and ABD095)^29^. Among these ABD derivatives, ABD094 showed the lowest sensitivity during T-cell proliferation assay^30^. High stability of these domains even after thermal or chemical denaturation may be due to the lack of cysteine amino acid residues and post translational modifications^27^.

Therefore, in the present study, GCSF was genetically fused to the ABD094 domain to study the possible structural and functional changes of GCSF protein in comparison to the commercially available GCSF molecules.

## Results

### Protein expression

**Figure 1:**
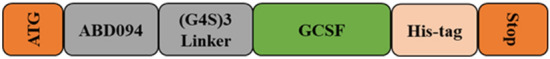
Schematic view of the expression cassette.

**Figure 2:**
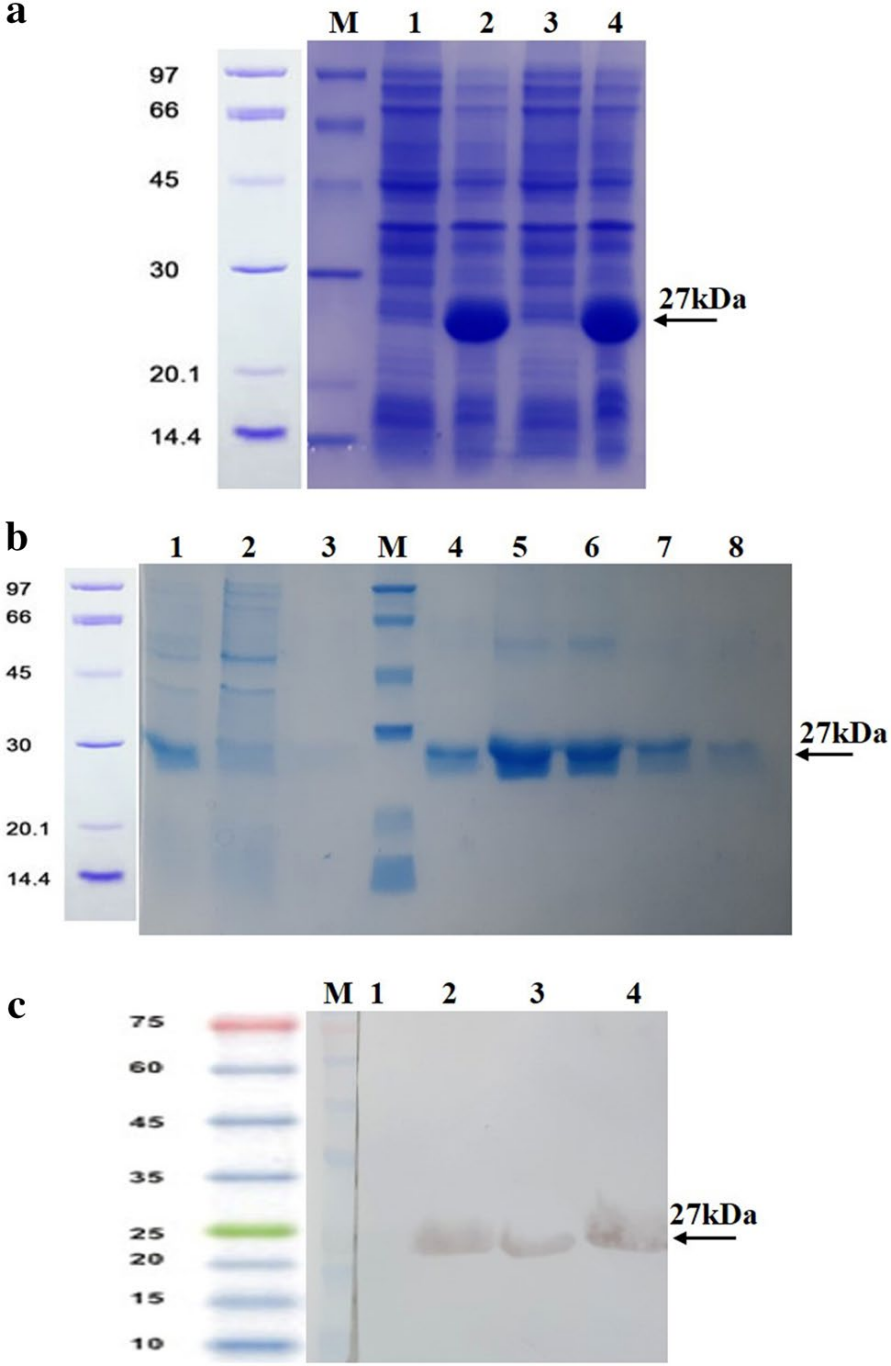
ABD-GCSF protein. **(a)** Protein expression: M: Protein Mw marker; #1, 3: Recombinant bacterial lysates before induction; #2, 4: Recombinant bacterial lysates after induction. **(b)** Protein purification: #1: Initial sample (IS); #2: Flow through (FT) sample; #3: Washing sample; M: Protein Mw marker; #4–8: Eluted samples. **(c)** Western blotting: M: Protein Mw marker; #1, 2: Bacterial lysates before and after induction; #3, 4: Eluted proteins. The full-size original gels and the blot are presented in supplementary Fig. S1a,b,c, respectively.

The expression cassette (Fig. 1) was subcloned in pET28a expression vector and confirmed by restriction digestion and sequencing (data not shown). The optimum expression level of the fusion protein was achieved at 30 °C using 0.25 mM IPTG 6 h post-induction (Fig. 2a and Supplementary Fig. 1a) which was nearly 60% of the total bacterial protein content. The recombinant protein (25.46 kDa) was successfully purified using Ni-agarose resin (Fig. 2b and Supplementary Fig. 1b) yielding to an 8 mg/l of bacterial medium and was confirmed by Western blotting with anti-His tag antibody (Fig. 2c and Supplementary Fig. 1c). According to the endotoxin assessment by Pyrotell test, LPS content of the purified ABD-GCSF protein was lower than the detection limit of the kit (< 0.25EU/ml) when 2EU/mg LPS of the Filgrastim (Ph. Eur. Method 2.6.14) was set as endotoxin limit of this recombinant GCSF derivative.

### Confirmation of ABD-GCSF refolding

The obtained results from Ellman's assay showed that free sulfhydryl concentration in refolded ABD-GCSF protein was similar to the Filgrastim molecule which is assumed to be a native refolded GCSF protein. Optical densities revealed that the number of thiol residues within unfolded ABD-GCSF are more than its refolded form after dialysis which indicated that cysteine residues were participated in the formation of correct disulfide bonds (Supplementary Fig. 2). In addition, a computer generated 3-D image of ABD-GCSF protein represented no free cysteine residue on the surface of the protein (Supplementary Fig. 3a,b). This observation could explain the unchanged optical absorbance of the Ellman’s reagent in the case of refolded ABD-GCSF after dialysis.

### Size exclusion chromatography (SEC)

**Figure 3:**
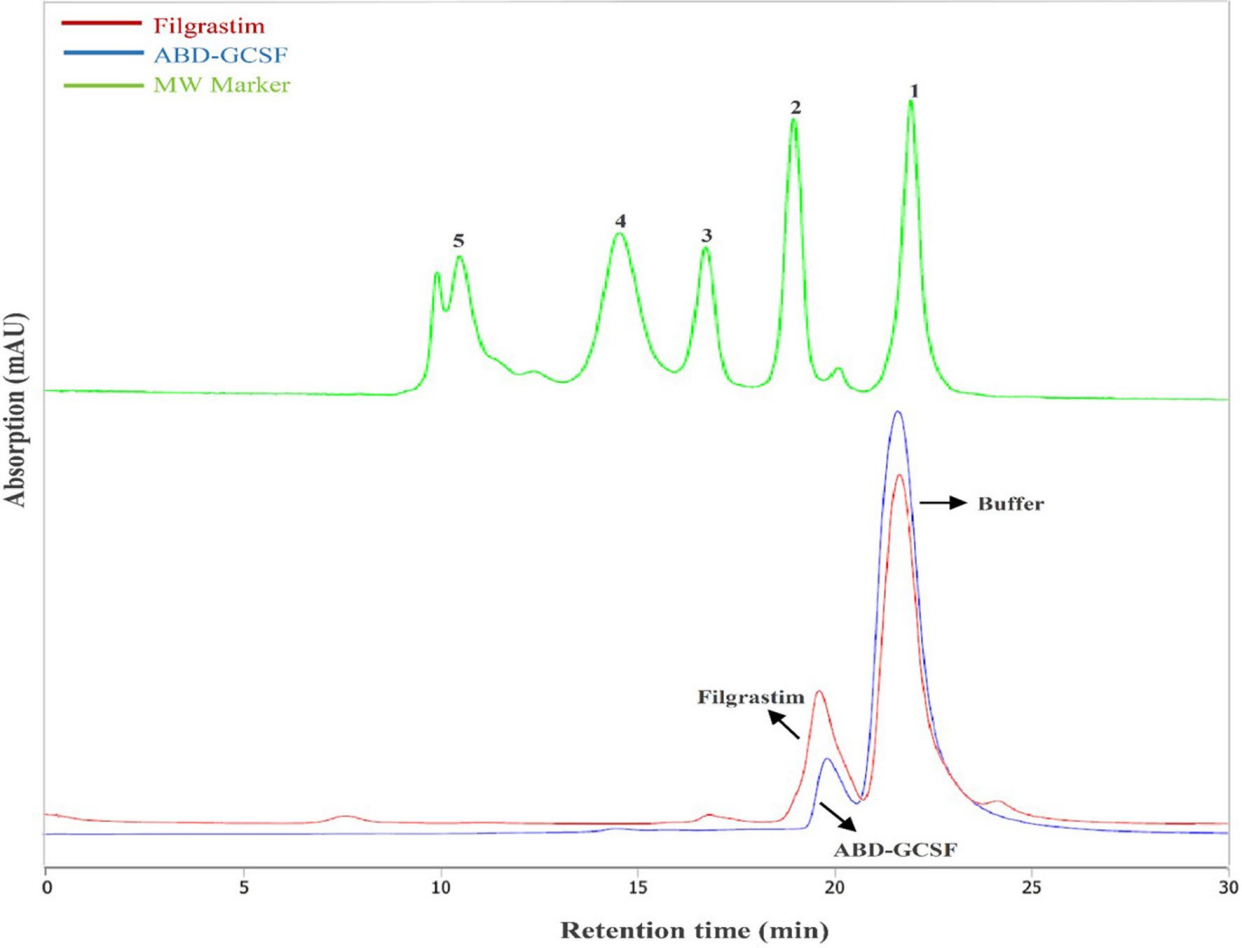
Size exclusion chromatography of Filgrastim and ABD-GCSF. Molecular weight marker includes the following proteins: (1) vitamin B12 (1.350 kDa); (2) horse myoglobin (17 kDa); (3) chicken ovalbumin (44 kDa); (4) bovine c-globulin (158 kDa); (5) bovine thyroglobulin (670 kDa); (5). Last peak obtained at retention time of 22 min represents excipients from the dilution buffer.

Hydrodynamic volume of the protein is one of the most important parameters affecting its renal infiltration and half-life^11^. The mobility of monomeric forms of Filgrastim and ABD-GCSF was analyzed on a calibrated SEC-HPLC column. The observed molecular weights were higher than those deduced from corresponding amino acid sequences which may be due to the ability of SEC column in separation of proteins based on their shape (hydrodynamic radius) as well as molecular weight^31^. SEC-HPLC results demonstrated that the addition of ABD to the GCSF protein almost did not change its hydrodynamic volume (Fig. 3). The obtained chromatogram revealed that there are no major impurities including truncated or aggregated forms of ABD-GCSF surrounding the main peak (monomer form) of the molecule which was appeared in retention time of about 19 min which is little different from Filgrastim control sample due to their molecular weight difference. The peak eluted at 22 min is derived from the sample dilution buffer.

### Dynamic light scattering measurements

**Figure 4:**
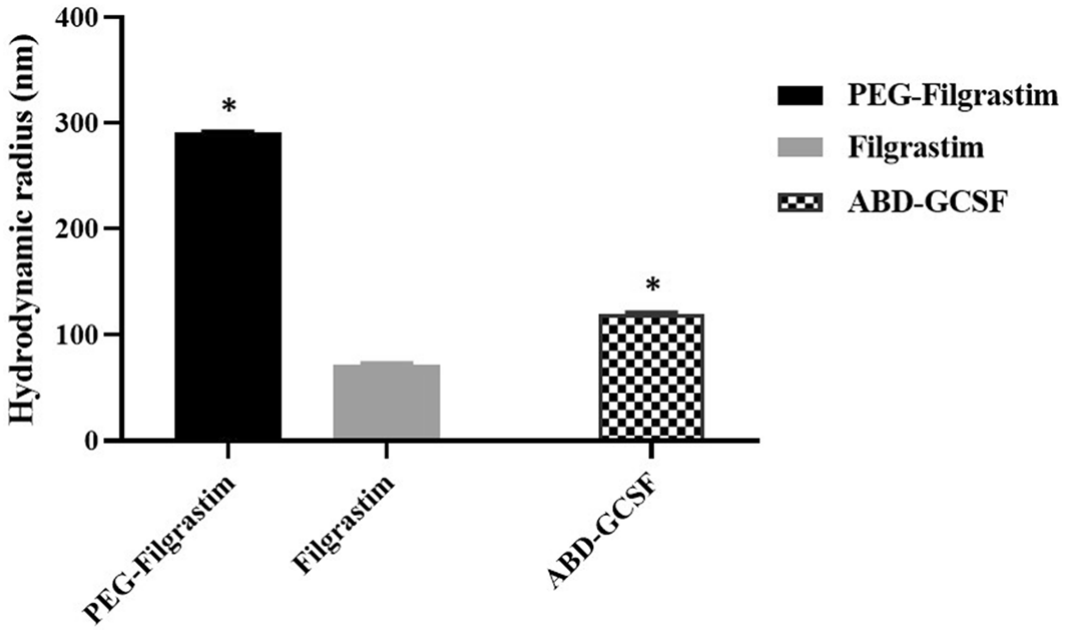
Dynamic light scattering measurements of ABD-GCSF and PEG-Filgrastim in comparison with Filgrastim. Data presented as mean ± SD. *Considered *P* values less than 0.05 in comparison to Filgrastim protein.

To assess the possibility of aggregation formation within the recombinant fusion protein and to determine the effect of ABD on GCSF hydrodynamic radius, DLS experiments were performed. Based on calculated PdI index (PdI: 0.48) and DLS graph (Supplementary Fig. 4), aggregation of ABD-GCSF was not significant. It was also shown that ABD fusion to the GCSF molecule could significantly increase the hydrodynamic radius of GCSF in comparison with Filgrastim (Fig. 4).

### Intrinsic fluorescence measurements

**Figure 5:**
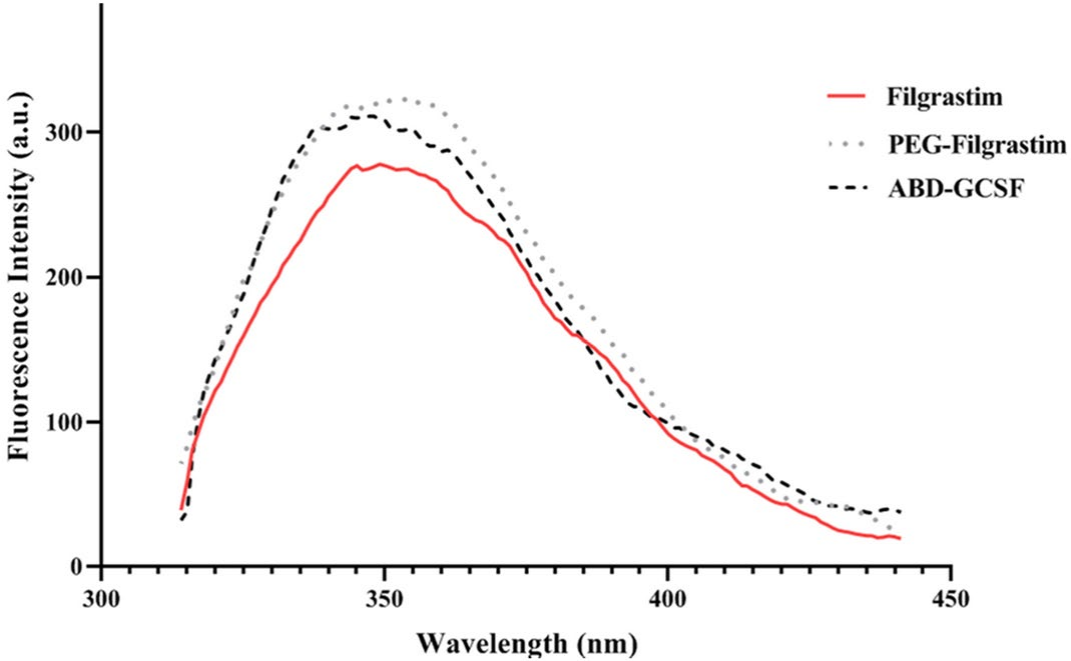
Fluorescence emission spectra of GCSF derivatives.

Following excitation and emission of tryptophan residues (Trp59 and Trp119) within GCSF protein at 295 nm and 300–450 nm, fluorescence spectra represented a similar maximum peak at 350 nm confirming the unchanged structural conformation of GCSF molecule by the fusion of ABD moiety (Fig. 5).

### Circular dichroism measurements

**Figure 6:**
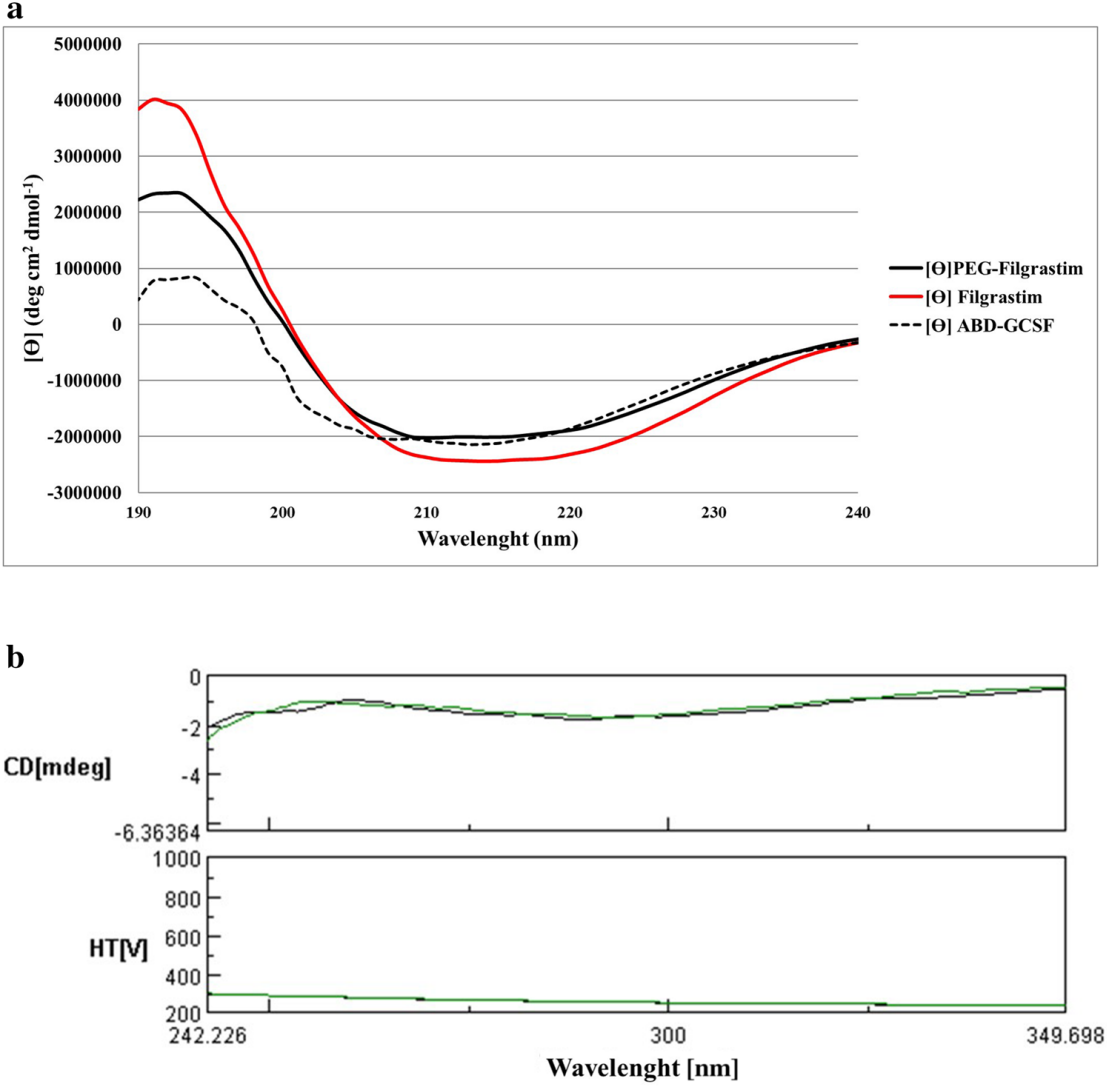
CD spectra of GCSF derivatives. **(a)** Far-UV spectrum (190–250 nm). **(b)** Near-UV spectrum (250–350 nm). Filgrastim and ABD-GCSF are represented in green and black lines, respectively.

Far and near-UV CD spectra have been shown in (Fig. 6a,b). In far-UV spectra, a positive peak at 195 nm and two minima at 208 and 222 nm were observed representing α-helical structures. According to the acquired near-UV spectra, it was concluded that three proteins had similar tertiary structures (Fig. 6b).

### Cell proliferation assay

**Figure 7:**
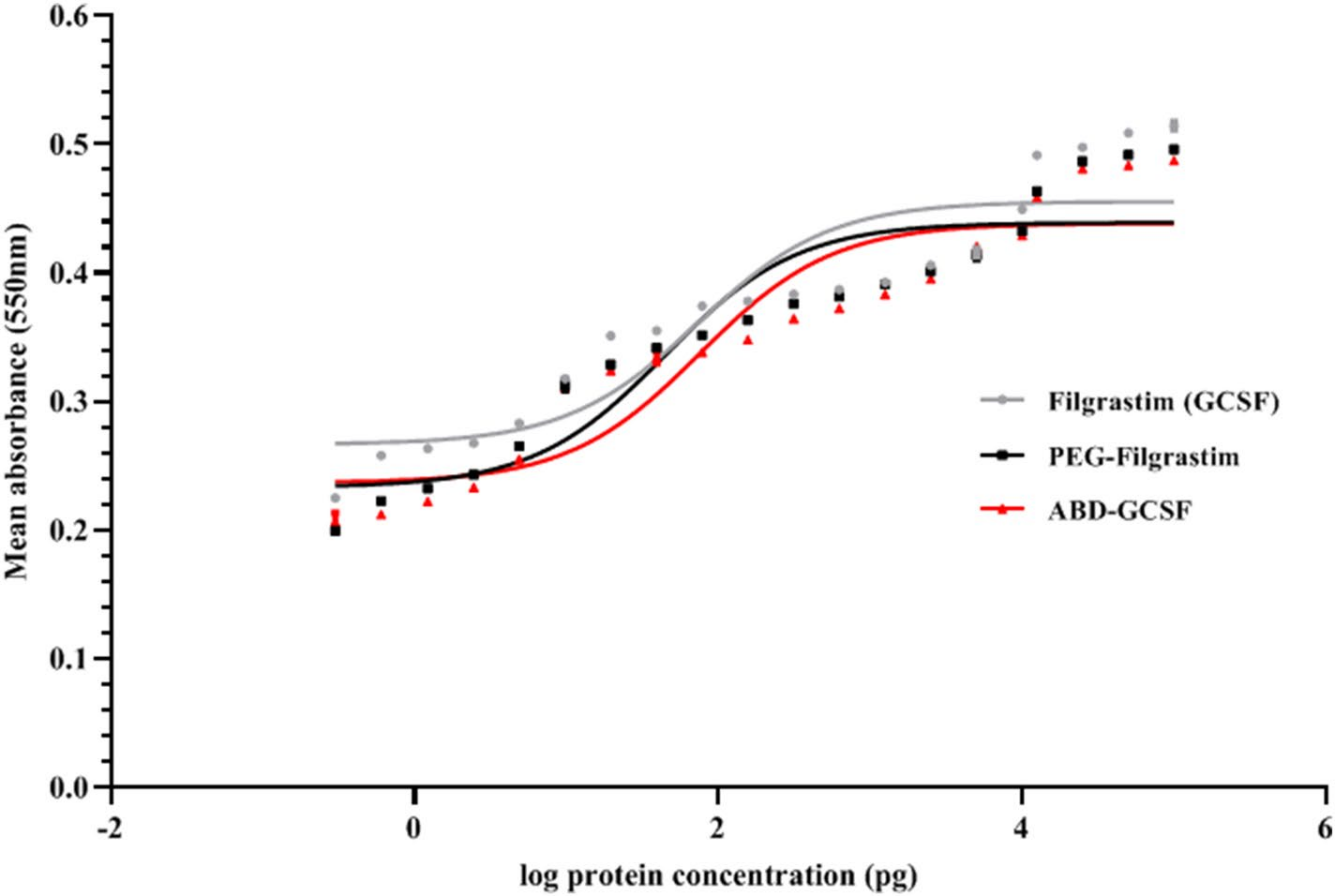
Dose response curves for GCSF derivatives on NFS-60 cells. Data are given as mean ± SD values of triplicate wells.

Proliferation of NFS-60 cell line depends on the presence of GCSF as a growth factor. The cells were treated with dilutions of Filgrastim, PEG-Filgrastim and ABD-GCSF for 72 h. Stimulus response graphs and the corresponding calculated EC50 values of Filgrastim, PEG-Filgrastim and ABD-GCSF were 73.1, 44.6, and 75.76 pg/ml (Fig. 7).

### Affinity of ABD-GCSF towards human serum albumin

**Figure 8:**
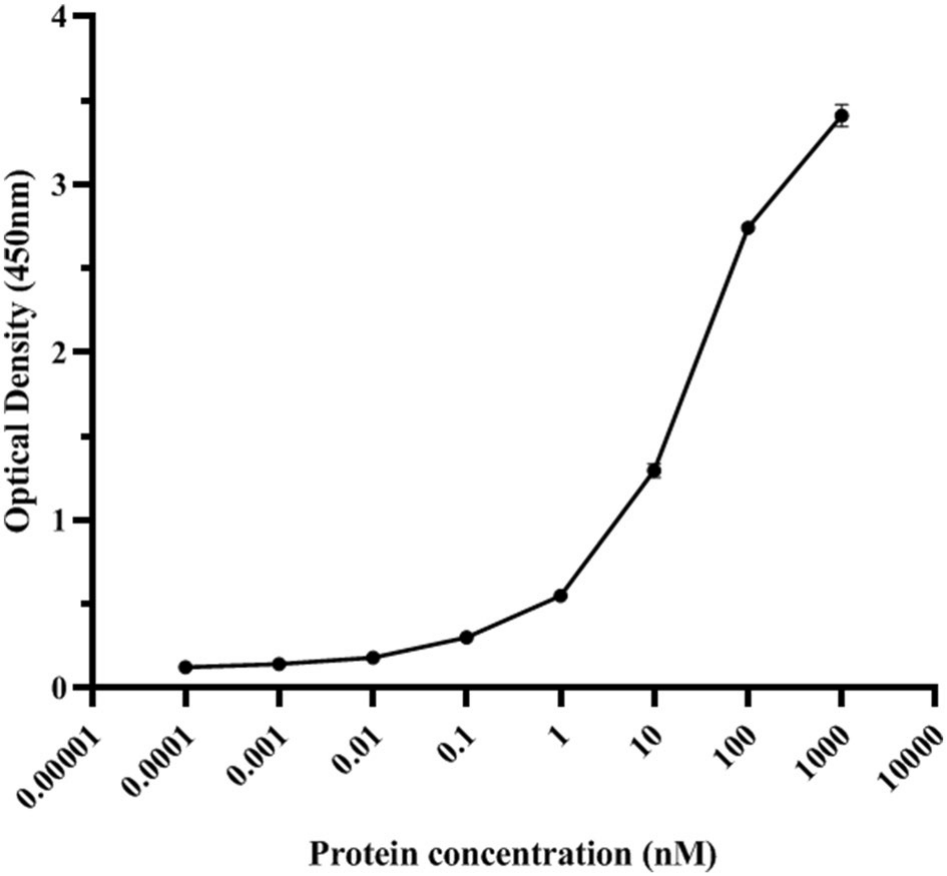
ELISA showing HSA binding of ABD-GCSF. The graph represents the mean ± SD of duplicates.

In a home-made ELISA assay the affinity of refolded ABD-GCSF protein for HSA was observed (Fig. 8) which can confirm the theoretical role of this domain in increasing serum half-life of the fusion protein through binding to the serum albumin.

### Animal studies

**Figure 9:**
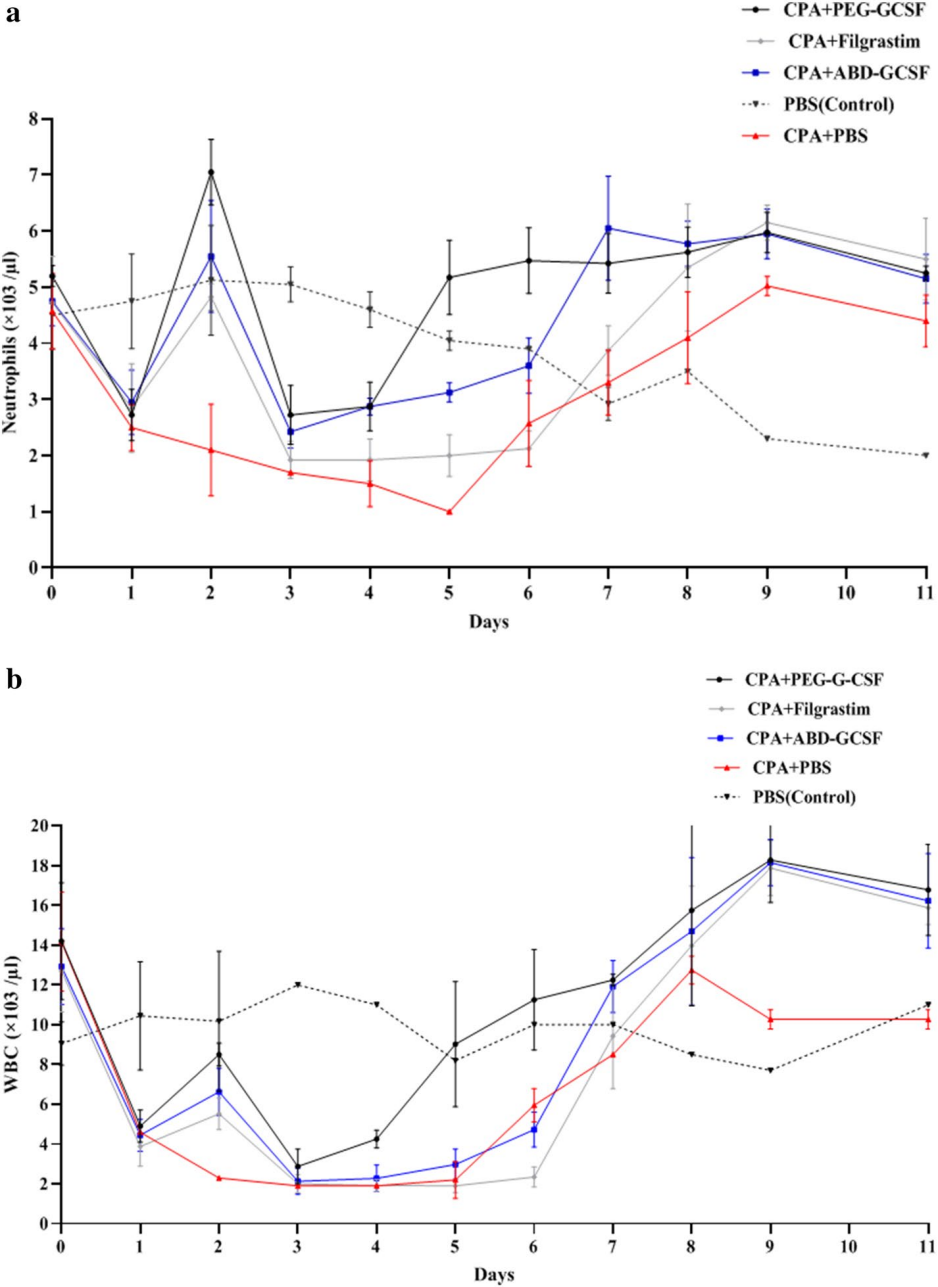
Mean of **(a)** neutrophil and **(b)** WBC counts in neutropenic rats after receiving single doses of GCSF derivatives. Data are means ± SE of 3 random rats/group.

To investigate pharmacodynamic and pharmacokinetic parameters of ABD-GCSF protein, 100 µg/kg of GCSF derivatives were injected to the rats one day after inducing neutropenia except PBS control group (Group 1). As illustrated in (Fig. 9), CPA-induced reduction of neutrophils and total WBCs was gradually improved from day 6 by the administration of GCSF derivatives especially within the rats received PEG-Filgrastim but these molecules could not increase red blood cells (RBC), lymphocytes and monocytes (Supplementary Fig. 5a,b,c).

**Figure 10:**
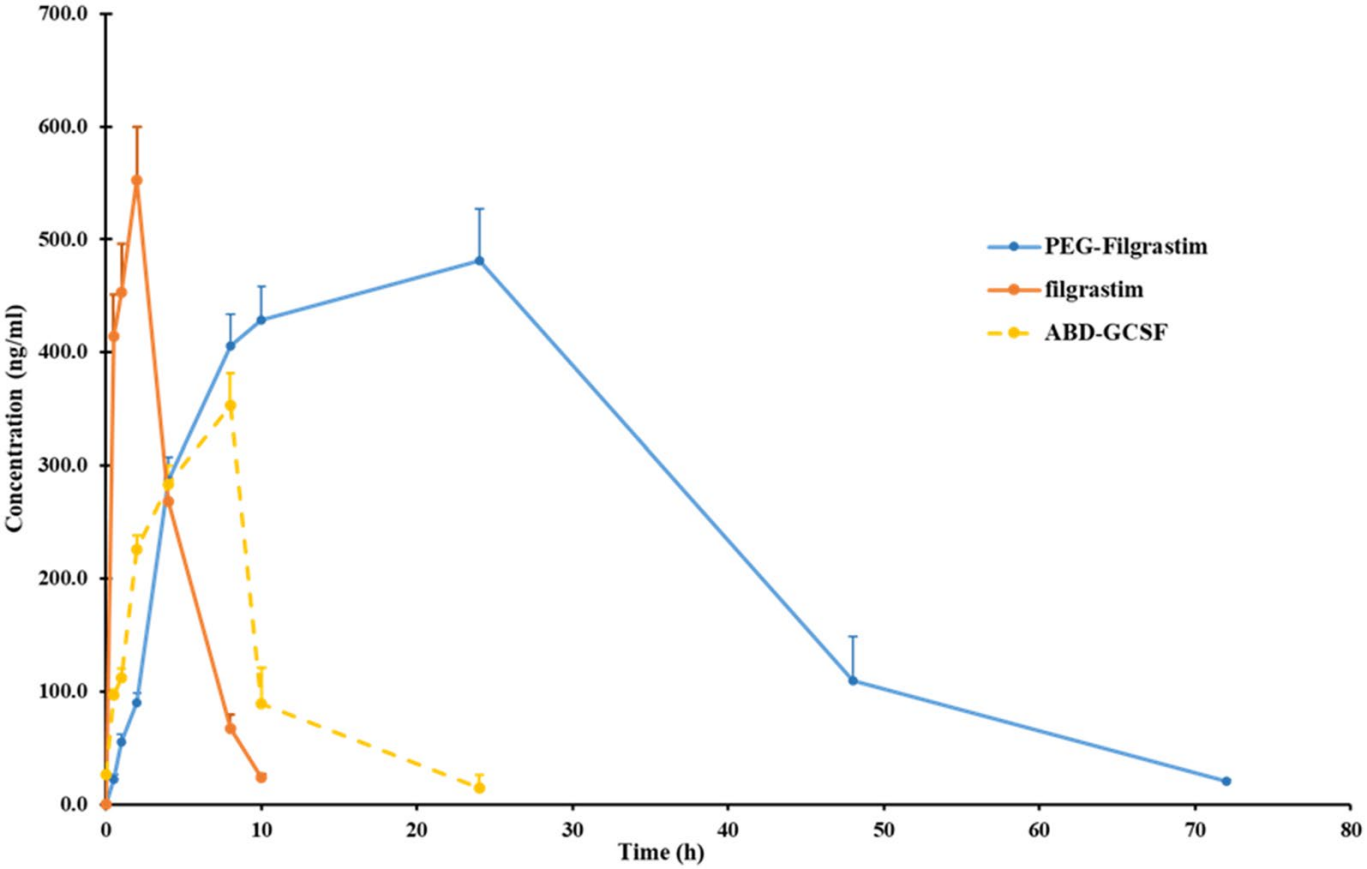
Plasma concentration–time profile of Filgrastim, PEG-Filgrastim and ABD-GCSF in rats. 5 rats/group subcutaneously received GCSF derivatives. All points report the mean ± SD of animals.

Based on ELISA results, the time points in which 50% of the proteins were eliminated (t_1/2_) were calculated as 1.7 ± 0.1, 10.0 ± 0.5 and 9.3 ± 0.7 h for Filgrastim, PEG-Filgrastim and ABD-GCSF, respectively. The lowest rate of protein clearance rate was calculated for PEG-Filgrastim which was significantly different from Filgrastim and ABD-GCSF (Table 1). On the other hand, subcutaneous administration of each protein resulted in maximum concentrations at 2, 24 and 10 h for Filgrastim (552.3 ± 47.7 ng/ml), PEG-Filgrastim (481.7 ± 45.2 ng/ml) and ABD-GCSF (353 ± 32 ng/ml). Calculated area under the protein concentration–time curves (AUC) suggested that ABD fusion to GCSF may improve the protein concentration within body in comparison with Filgrastim (Table 1). Filgrastim, PEG-Filgrastim, and ABD-GCSF were cleared from blood samples 10, 96 and 24 h post-injection according to the limit of quantification (LOQ) index of human GCSF ELISA kit (Fig. 10). Mean residence time (MRT) values were estimated to be 3.4 ± 0.1, 17.9 ± 2.2 and 9.9 ± 0.4 h for Filgrastim, PEG-Filgrastim and ABD-GCSF, respectively. Calculated AUC_0-∞_ value of Filgrastim (2315.33 ± 129.3 ng.h/ml) showed that maintenance of Filgrastim within the body is lower than ABD-GCSF and PEG-Filgrastim (6232.1 ± 531.5and 16,773.8 ± 2371.8 ng.h/ml, respectively) representing the decreased total clearance rates of GCSF derivatives (Filgrastim: 43.3 ± 2.3; PEG-Filgrastim:6.056 ± 0.8341; ABD-GCSF: 16.1 ± 1.4 ml/kg.h, respectively).

**Table 1:** Major pharmacokinetic parameters of administered ABD-GCSF in a neutropenic rat model.

Proteins	T_max_ (h)	C_max_ (ng/ml)	AUC _(0-t)_ (ng h/ml)	AUC _(0-∞)_ (ng h/ml)	K_a_ (h^−1^)	K_e_ (h^−1^)	t_1/2_ (h)	CL/F (ml/h/kg)	MRT (h)
Filgrastim	2.0 ± 0.0	552.3 ± 47.7	2406.6 ± 218.0	2315.3 ± 129.3	1.22 ± 0.11	0.4 ± 0.00	1.7 ± 0.1	43.3 ± 2.3	3.4 ± 0.1
PEG-Filgrastim	24.0 ± 0.0*	481.7 ± 45.2*	16,773.8 ± 2371.8*	16,773.8 ± 2371.8*	0.103 ± 0.001	0.07 ± 0.00*	10.0 ± 0.5*	6.056 ± 0.83*	17.9 ± 2.2*
ABD-GCSF	10.0 ± 0.0*	353 ± 32.0*	6038 ± 520.7*	6232.1 ± 531.5*	0.187 ± 0.004	0.08 ± 0.0*	9.3 ± 0.7*	16.1 ± 1.4*	9.9 ± 0.4*

Data is represented as the means ± SD of 5 rats/group.

*T*_*max*_, Time to reach the peak plasma concentration; *t*_*1/2*_, Elimination half-life; *C*_*max*_, Maximum observed plasma concentration; *K*_*e*_, Elimination rate constant; *CL/F*, Apparent total clearance; *AUC*, Area under the serum concentration–time curve; *MRT* Mean residence time.

*P < 0.05 in comparison with the Filgrastim group.

## Discussion

Although GCSF molecule is commonly used to reduce the neutropenic side effects of myeloid suppressive anticancer drugs^32^ its rapid clearance has forced development of several strategies to improve its pharmacokinetic properties^12,32,33^ Increasing the GCSF size by fusing the protein to another protein or to a polymer such as polyethylene glycol (PEG) was one of the options. For this purpose, several studies have been conducted to see if N or C terminal amino acids of this molecule can be modified. In the case of PEG-Filgrastim, the long-acting Filgrastim, it was confirmed that the attachment of a single linear 20 kDa PEG molecule to the N-terminal Methionine can not significantly interfere with GCSF/GCSF-R interaction^34^. Previous studies have shown that the attachment of albumin binding domains (ABDs) could enhance protein half-life^24,25,28^. For example, this domain has been genetically fused to Exenatide and improved its short half-life (30 min to 16 h)^25^. Serum stability of an anti-cancer bispecific diabody was also extended five–six fold by the fusion of ABD (10 vs. 64 min)^24^. Therefore, in the present study ABD peptide with improved stability and safety (ABD094)^24,25,27,29^ was genetically attached to the N-teminus of the Filgrastim molecule by the use of a flexible Gly_4_Ser linker peptide of 15 amino acids in order to have the minimum structural change of GCSF/GCSF-R interface. Also six histidine residues were inserted at upstream of ABD-GCSF gene in order to be purifed with Ni–NTA affinity column. It has been confirmed that His-tag does not change the expression level, solubility, folding and biological function of the recombinant proteins due to its small size^35,36^.

According to the previous studies, *Escherichia coli* can be considered as a suitable expression host cell in comparison with mammalian cells due to its short doubling time, well-known genome, easy scale-up and low cost of growth conditions^37–39^. Although many studies have revealed that *E. coli* can be selected as the expression host for the proteins which have no complex post translational modifications (PTM)^39,^ disulfide bond formation and appropriate refolding can be challenging issues which should be considered for *E. coli* expression systems. In the case of GCSF molecule, development of Filgrastim and its biosimilar forms which are produced in *E. coli* suggest this host as a suitable expression system.

Size exclusion HPLC chromatography (SEC) showed that multimeric forms of ABD-GCSF were not significant confirming the good quality of the dialyzed recombinant protein. Complete removal of the denaturant agent and correct refolding of the protein was further approved by DLS PdI index and SDS-PAGE (Supplementary Fig. 6). Tryptophan, phenylalanine and tyrosine residues usually contribute in intrinsic fluorescence properties of proteins^40^. Based on tryptophan fluorescence properties, intensity of this amino acid could predict the tertiary structure of the proteins. In the case of PEG-Filgrastim, the increased fluorescence intensity may be due to the Trp relocation towards outside of PEG-fused GCSF confirming previously published results^41^. The obtained IFS spectra of ABD-GCSF protein did not represent any significant shift confirming unchanged structural conformation of the protein in comparison with the commercially available GCSF proteins. In addition, the increased height of the peaks for PEGylated and ABD-GCSF could interpret the buried tryptophan residues of Filgrastim causing a more compacted structure of this molecule. Also, in Far-UV spectrum of ABD-GCSF, 2 minima peaks at 208 and 222 nm and one positive peak at 195 nm were observed which were similar to Filgrastim. CD spectra of ABD-IFNα and ABD-Ciliary neurotrophic factor (CNTF) also confirmed that ABD could not change secondary structure of these molecul^15,42^.

Herein, on molar basis according to the molecular weight of protein monomers, the calculated EC50 values represented 2.83, 3.89 and 1.14 µM for Filgrastim, PEG-Filgrastim and ABD-GCSF, respectively. The results are in accordance to the previously published data of GCSF-Fc^12^, GCSF-transferin^43^and PEG-GCSF^12,44,45^.

In the present study neutropenic rats were used to evaluate biological activity of recombinant ABD-GCSF in analogy between two commercial forms. Any changes in pharmacodynamic curves strongly depends on potency of the administered drug in reduction of CPA side-effects. It was also revealed that duration of neutropenia in rats received PEG-Filgrastim and ABD-GCSF was shorter than the group received Filgrastim which may be due to their prolonged blood circulation. To get more detailed view, clinical effects (PD) should be studied along with pharmacokinetics properties (PK). Previous studies have shown that the efficacy of GCSF on inducing neutrophil production depends on its serum concentration and total clearance rate^46–48^. Plasma concentration–time profile of ABD-GCSF protein was wider than Filgrastim which resulted to a prolonged T_max_ (10.0 ± 0.0 h for ABD-GCSF vs. 2.0 ± 0.0 h for Filgrastim) representing different clearance patterns and confirmation of more efficiency of ABD-GCSF in stimulation of neutrophils compared to the Filgrastim-treated group. On the other hand, different maximum concentration (C_Max_) of ABD-GCSF protein (63.91% of Filgrastim; 353 ± 32.0 vs. 552.3 ± 47.7 ng/ml) could suggest different pathways in protein absorption^49,50^. In contrast, plasma half-life and C_Max_ of PEG-Filgrastim (10.0 ± 0.5 h, 481.7 ± 45.2 ng/ml) was more than ABD-GCSF (9.3 ± 0.7 h, 353 ± 32.0 ng/ml) and Filgrastim (1.7 ± 0.1 h, 552.3 ± 47.7 ng/ml) which could be explained by its hydrated polyether chain preventing protease degradation and macrophage uptake of the molecule. Consequently, its total clearance rate (6.056 ± 0.8341 ml/h.kg) was lower than ABD-GCSF and Filgrastim (16.1 ± 1.4 and 43.3 ± 2.3 ml/h.kg). As a result, maximum neutrophil count in PEG-Filgrastim group was higher than two other groups which decreased neutropenic days in these rats. The calculated time for reaching to the maximum concentration (T_Max_) of PEG-Filgrastim and ABD-GCSF (24 vs. 10 h) could refer to their hydrodynamic radius and/or lymphatic absorption which result to slower release of the molecule after s.c adminstration^49,50^. According to the previous studies, elimination of GCSF is mostly through non-renal clearance by the neutrophils^12,51–54^. In brief, GCSF binds to its receptors mainly located on the surface of peripheral neutrophils follows with internalization and degradation of GCSF protein^51,52,55^. Therefore, GCSF serum concentration will be mainly associated to the number of neutrophils and the other cells possessing GCSF receptor especially in bone marrow and spleen^54^. In the present study, higher AUC (0-∞) value of ABD-GCSF (6232.1 ± 531.5 ng.h/ml) in comparison to Filgrastim (2315.3 ± 129.3 ng.h/ml) indicated the improved ABD-GCSF plasma circulation. This phenomenon was supported by the confirmed affinity of ABD-GCSF towards HSA in a home-made ELISA assuming recycled protein through FcRn with reduced lysosomal degradation and kidney uptake. On the other hand, plasma half-life of ABD-GCSF could be associated with its albumin attachment characteristics and reduced cellular internalization^21,51^. The obtained pharmacokinetic indices of PEG-Filgrastim are in accordance with previous studies in which a single dose of PEG-Filgrastim act as effective as multiple doses of Filgrastim because of its increased hydrodynamic radius^56,57^. Altogether, total clearance rates, time of obtaining GCSF plasma peak (24 h) and AUC _(0-∞)_ values can suggest that PEG-Filgrastim is more stable than ABD-GCSF. The observed improved plasma circulation of ABD-GCSF confirmed previously published studies on this domain. The short serum half-life of IFN-α was increased by genetically fusion of ABD to its C and/or N terminus (from 3 to 19.3 and 32.8 h; 6.5 and 11-fold)^15^. In another study, the attachment of HER2-binding affibody to ABD035 could increase its half-life to 80-fold (0.5 vs. 41 h)^28^. ABD fusion to IL-2 decreased clearance rate (from 176.4 ± 4.0 to 77.4 ml/h/kg) while increased serum half-life (46 vs. 150 h, 3 fold)^58^. In the case of doxorubicin, the chemotherapy drug, the clearance rate enhanced from 4.38 to 23 h (five fold) after its attachment to ABD^59^. Serum half-life of a receptor blocking anticalin protein (OX40 Ac) could increase from 0.5 to 60 h (12-fold) by genetically fusion to ABD094 in a mouse model^60^. In another study, N-terminal ABD035 could extend half-life of ciliary neurotrophic factor (CNTF) from 34 to 483 min (14-fold)^42^. Low serum half-life of recombinant hTRAIL was also enhanced from 0.32 to 14.1 h (28-fold) by the fusion of ABD035 to the N-terminus. Their results showed that hTRAIL-ABD format could not efficiently attach to the albumin and no change of circulation half-life of the hTRAIL was observed^61^. This finding supports that fusion of ABD moiety to the N or C terminus of the candidate protein should be studied carefully to achieve the longer serum half-life.

## Conclusions

Our results showed that attachment of ABD moiety could increase circulating half-life and stability of GCSF protein without affecting its hematopoietic characteristics. It seems that genetically fusion of safe polypeptides like ABD to the small drug proteins may have promising results in development of biobetter biologics.

## Methods

### Ethics statement

All experiments and procedures were approved by the Ethics Committee of Pasteur Institute of Iran (IR.PII.REC.1399.013) and performed in accordance with the approved guidelines and regulations.

### Expression cassette

GCSF (Filgrastim) encoding amino acid sequence was extracted from drug bank (Accession No. DB00099). ABD094 amino acid sequence compromising of 46 amino acids (Mw: ~ 5.8 kDa) was obtained from published patents (US10206975B2 and JP2014557602A). The two amino acid sequences were fused using a flexible Gly_4_Ser linker peptide compromising 15 amino acids ((G_4_S)3). A histidine tag was designed at upstream of the ABD-GCSF gene cassette (Supplementary Fig. 7), synthesized after *E. coli* codon optimization and subcloned into pET28a expression vector (Novagen, USA) at *NcoI* and *HindIII* restriction sites.

### Protein expression

The expression of recombinant protein was induced in *E. coli* BL21 (DE3) (Novagen, USA) host cells. Luria–Bertani (LB) broth medium supplemented with kanamycin 30 µg/ml was inoculated with the recombinant bacteria and incubated at 37 °C shaker incubator until optical density of the medium reached 0.5 at 600 nm. Isopropyl β-d-1-thiogalactopyranoside (IPTG) was used as expression inducer (0.25 mM) and bacteria were incubated for further 6 h at 30 °C. Bacterial pellet was collected by centrifugation at 9000 rpm for 3 min and protein expression level was quantified on 12% SDS-PAGE stained with Coomassie Brilliant Blue G250 dye.

Bacterial lysates were run on 12% SDS-PAGE and transferred to nitrocellulose membrane (Amersham, UK) in a semi-dry transfer system (Bio-RAD) (18v, 25 min). After overnight (o/n) blocking the membrane with 2% (w/v) skim milk in phosphate buffered saline (PBS) at 4 °C, 1:2000 dilution of Horse Radish Peroxidase (HRP) conjugated anti-His antibody (Sigma, USA) was added to the membrane for 2 h at room temperature (RT). The membrane was washed 4 times with PBS/Tween-20 (0.05%) and the corresponding His-tagged fusion protein band was visualized using 3,3’-diaminobenzidine (DAB) substrate (Sigma, USA).

### Protein purification

To purify the recombinant protein, colonies was inoculated into 500 ml LB broth medium under the above-mentioned culture condition. Bacterial pellet was resuspended in lysis buffer I (50 mM NaH_2_PO_4_, 10 mM Imidazole, 300 mM NaCl; pH8.0) and sonicated by 20 pulses (20 s with the same interval time) and centrifuged at 10,000 rpm for 20 min at 4 °C. The pellet was resuspended in lysis buffer II (50 mM NaH_2_PO_4_, 300 mM NaCl, 10 mM Imidazole, 8 M Urea; pH8.0) and sonicated for further 3 pulses (20 s/pulse) and centrifuged again. The supernatant was filtered through 0.45 µm syringe filter and was loaded to the Ni-agarose resin (ABT Agarose Bead Technologies, Spain) under denaturing conditions. The column was washed with 30 volume of washing buffer I (50 mM NaH_2_PO_4_, 300 mM NaCl, 30 mM Imidazole, 8 M Urea, 0.1% Triton-X114; pH8.0) to remove weakly bounded proteins and bacterial lipopolysaccharides (LPS) followed by the second washing step (50 mM NaH_2_PO_4_, 300 mM NaCl, 30 mM Imidazole, 8 M Urea, pH8.0) to remove residual Triton-X114. The recombinant protein was eluted by the elution buffer (50 mM NaH_2_PO_4_, 300 mM NaCl, 250 mM Imidazole, 8 M Urea; pH8.0) at a flow rate of 1.5 ml/min.

Refolding of the eluted protein was performed through dialysis in order to gradually remove urea (from 8 M to 0). The dialyzed protein was finally kept in phosphate-buffer (PB) (8 mM Na_2_HPO_4_, 1 mM KH_2_PO_4_, 137 mM NaCl, 3 mM KCl; pH7.4). Protein concentration was performed using Centriprep-3 kDa (Amicon, USA) and the protein concentration was measured by NanoDrop™ 3000 spectrophotometer (Bio-RAD).

The pyrogenicity of the purified recombinant protein was quantified by Pyrotell gel clot LAL kit (USA; Sensitivity 0.25EU/ml of analyzed solution) according to the manufacturer's instruction. Briefly, Limulus Amebocyte lysate was incubated with serially diluted ABD-GCSF protein samples at 37 °C for 60 min and a positive test will be indicated by the formation of gel which does not collapse when the tube is inverted.

### DTNB colorimetric analysis (Ellman)

Ellman’s reagent, 5, 5’‐dithiobis‐(2‐nitrobenzoic acid) (DTNB), was used for quantification of free sulfhydryl groups in ABD-GCSF protein in comparison with Filgrastim. In brief, the reaction buffer (0.1 M sodium phosphate, 1 mM EDTA; pH8.0) containing 5 mM Filgrastim or different concentrations of ABD-GCSF (5, 3, 2, 1 mM) and DTNB was incubated at RT for 15 min. The change in optical absorbance was measured at 412 nm. The concentration of free thiol groups was measured using molar extinction coefficient of chromophore (1.415 × 10^4^ M^−1^ cm^−1^). The negative control sample was DTNB in the absence of any protein. Different concentrations (1.5, 1.25, 1.0, 0.75 and 0.25 mM) of cysteine amino acid solution were used as positive control.

### Size exclusion chromatography (SEC)

For comparing the hydrodynamic volume of commercially available GCSF protein (Filgrastim; Pooyesh Darou, Iran) and ABD-GCSF, size exclusion chromatography (SEC) was carried out according to the European Pharmacopoeia version 9.1. In brief, proteins were diluted in 0.06 M sodium acetate (pH4.0) to 200 µg/ml. In the next step, 8 µg of each protein was injected into TSK gel G3000SWxl column (Tosoh Bioscience, Japan) connected to a Shimadzu HPLC system (Kyoto, Japan) and eluted by an isocratic mobile phase of 0.03 M (NH_4_)_2_HPO_4_ (pH7.0) at a flow rate of 0.5 ml/min at 30ºC. The ultraviolet (UV) absorbance was recorded at 215 nm. Molecular weight of the proteins was also estimated based on the retention time of gel filtration standard protein (Bio-RAD, Cat No. 151-1901).

### DLS

Dynamic light scattering experiments were performed to determine the effect of ABD tag on hydrodynamic radius of the GCSF protein. In brief, 0.5 mg/ml of each protein (Filgrastim, PEG-Filgrastim manufactured by CinnaGen, Iran, and ABD-GCSF) was prepared in double distilled water (ddH_2_O) and analyzed by Zetasizer ZEN3600 system (Malvern, Germany) at 25 °C. All results were reported as hydrodynamic radius in nm.

### IFS

To investigate tertiary conformational changes, intrinsic fluorescence emissions of proteins were measured using Cary Eclipse Varian Spectrophotometer (Agilent Technologies, Germany) in emission wavelength ranging from 300 to 400 nm. 300 µg/ml concentration of the proteins was prepared in PBS (pH7.4) in 1 cm path length quartz cells. Fluorescence emission was excited at 295 nm. Excitation and emission slits of 5 and 10 nm were considered.

### CD analysis

For finding possible secondary structural changes of ABD-GCSF protein in comparison to the commercially available GCSF molecules (Filgrastim and PEG-Filgrastim), Circular dichroism (CD) analysis was performed using J-810 Spectropolarimeter (Jasco Instruments, Japan). In brief, 0.2 mg/ml protein in double distilled water (ddH_2_O) was exposed to far (190–260 nm) and near (250–320 nm) UV spectra within 1 mm path length quartz cell at 25 °C. CD spectra were recorded by the average of two scans, 1 nm band width and scanning speed of 500 nm/min.

### Cell proliferation assay

GCSF-dependent NFS-60 cells (murine myeloblastic cell line, Pasteur Institute of Iran) were tested in in vitro biological activity assessment of ABD-GCSF fusion protein in comparison with Filgrastim and PEG-Filgrastim. NFS-60 cells were cultured in RPMI-1640 medium supplemented with 10% fetal bovine serum (FBS), 0.025 mM sodium-pyruvate, 1% penicillin/streptomycin, 0.025 mM 2-ME and 33 IU/ml IL-3 (Sigma, USA). 5 × 10^3^ cells/well were seeded in 96-well plates and serially dilutions (0.0001, 0.001, 0.01, 0.1, 1, 10, 100, 1000, 10,000 ng/ml) of Filgrastim, PEG-Filgrastim and ABD-GCSF were added to the wells and incubated for 72 h. Culture medium alone was served as negative control. The cells were treated with 3-(4,5 dimethylthiazol-2-yl)-2,5-diphenyl tetrazolium bromide (MTT) (Sigma, USA) and incubated for further 4 h at 37 °C. 1% SDS was added to the wells and incubated for 16 h at 37 °C. Optical densities were measured at 550 nm using microplate reader spectrophotometer (BioTeK, USA). All tests were done in triplicate. EC_50_ values, the concentration of substrate in which 50% of maximum proliferation was achieved, were calculated by Prism software (v. 8.0).

### Albumin binding assay

A home-made ELISA assay was developed to measure the affinity of ABD-GCSF towards human serum albumin (HSA). In brief, 96-well plate was coated with HSA (1 µg/well) in carbonate-bicarbonate buffer at 4 °C (o/n). The plate was blocked with 2%w/v skim milk for 2 h at 37 °C. After washing step with PBS supplemented with 0.05% Tween-20, serial dilution of ABD-GCSF (0-1000 nM) was added to the wells and incubated for 2 h RT. HRP-conjugated anti-His antibody (1: 2000) was used as the secondary antibody. Assessment of binding reaction was done using 3,3',5,5'-Tetramethylbenzidine (TMB) substrate (Sigma, USA). H_2_SO_4_ was added to the wells to stop the reaction and optical density was measured at 450 nm by a microplate reader (BioTeK, USA).

### Pharmacodynamics

The effect of ABD-GCSF in acceleration of neutrophil count was investigated in normal Sprague Dawley male rats (6–7 weeks, 250–300 g weight). The research protocols and animal studies were approved by the Ethics Committee of Pasteur Institute of Iran (IR.PII.REC.1399.013) and followed ARRIVE reporting guidelines^62^. Animals were adopted to the conditions of light and humidity for one week and were randomly divided into five groups (five rats/group). The animals except control group (Group 1) intraperitoneally (*i.p*) received 100 mg/kg of cyclophosphamide (CPA) for induction of neutropenia on day zero^12,63^. On day 1, the rats in Groups 2 to 4 subcutaneously (*s.c*) received 100 µg/kg of Filgrastim, PEG-Filgrastim, or ABD-GCSF, respectively and the 5^th^ group received PBS alone as control^12,30,64^. Blood samples were collected from tail vein in non-vacuumed K_2_EDTA Nex tubes (Nexamo Technoplast, India) according to the designed time schedule on 0, 1, 2, 3, 4, 5, 6, 7, 8, 9 and 11 days post GCSF injection. Complete blood counting (CBC) of neutrophils, lymphocytes, eosinophils, monocytes, red and white blood cells was done by hematology analyzer Celltac alpha (Nihon Kohden, Japan).

### Pharmacokinetics

To determine the basic pharmacokinetic parameters of Filgrastim, PEG-Filgrastim and recombinant ABD-GCSF, plasma samples were used to quantitate GCSF concentration in the above-mentioned rat groups using human Quantikine GCSF ELISA kit (R&D Systems, USA). Briefly, blood samples were collected at 0, 0.5, 1, 2, 4, 8, 10, 24, 48, 76, 92, 120, 144, 168, 192 and 240 h post *s.c* drug injection and centrifuged at 3,000 rpm for 15 min to separate plasma. GCSF level was measured using the kit. Pharmacokinetic parameters were calculated using the log linear trapezoidal method. Half-life (t_1/2_), maximum concentration (C_max_), time of maximum concentration (T_max_), apparent total clearance rate (CL/F), area under the plasma concentration–time curve (AUC) and AUC from time zero to the time that protein is detectable in the blood (AUC_0-t_), AUC up to unlimited time (AUC_0-∞_), AUC up to the last measurable concentration (AUMC_0-t_), and mean residence time (MRT) were calculated. Linear regression of terminal log-linear phase and residual method were respectively used for obtaining of terminal rate constant (K_el_).

### Statistical analysis

GraphPad Prism (v. 8.0) and SPSS software (v. 21) were used. All data were reported as mean ± SD. *P* values less than 0.05 were considered significant.

## Supplementary Information

Supplementary Information.
