# Supplementary material for: Extension of human GCSF serum half-life by the fusion of albumin binding domain
Source: Sci Rep. 2022 Jan 13;12:667. doi: 10.1038/s41598-021-04560-6 (PMC8758692; doi:10.1038/s41598-021-04560-6)
Supplement: Supplementary file 1 — Supplementary Information. [file 41598_2021_4560_MOESM1_ESM.docx]

**Extension of Human GCSF serum half-life by the fusion of albumin binding domain**

Fatemeh Yadavar Nikravesh^1^, Samira Shirkhani^1^, Elham Bayat^1^, Yeganeh Talebkhan^1*^, Esmat Mirabzadeh^2^, Masoumeh Sabzalinejad^1^, Hooman Aghamirza Moghim Aliabadi^1^, Leila Nematollahi^1^, Yalda Hosseinzadeh Ardakani^3^*, Soroush Sardari^1^

^1^ Biotechnology Research Center, Pasteur Institute of Iran, Tehran, Iran.

^2^ Department of Molecular Medicine, Pasteur Institute of Iran, Tehran, Iran.

^3^ Biopharmaceutics and Pharmacokinetic Division, Department of Pharmaceutics, Faculty of Pharmacy, Tehran, Iran.

**Supplementary Figure S1a.** **ABD-GCSF protein.** The full size original image of Figure 2a: #1, 2: Lysate of empty *E. coli* BL21 (DE3) cells before and after induction (BI, AI); #3: Lysate of *E. coli* BL21 (DE3) harboring pET28a vector after induction; M: Protein Mw marker (Fermentas); #4, 6: Recombinant *E. coli* lysate before induction (BI); #5, 7: Recombinant BL21 *E. coli* lysate after induction (AI).


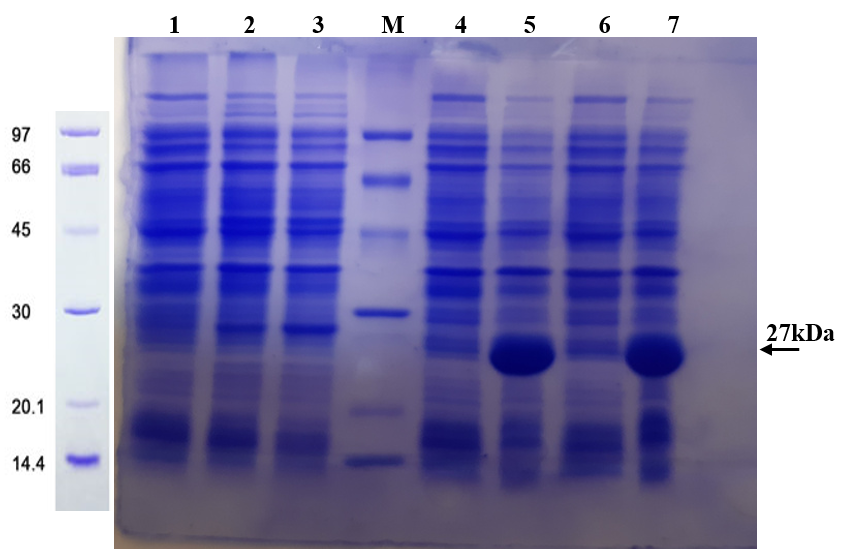


**Supplementary Figure S1b.** **ABD-GCSF protein.** The full size original image of Figure 2b: #1: Initial sample (IS); #2: Flow through (FT) sample; #3: Washing sample; M: Protein Mw marker; #4-8: Eluted samples.


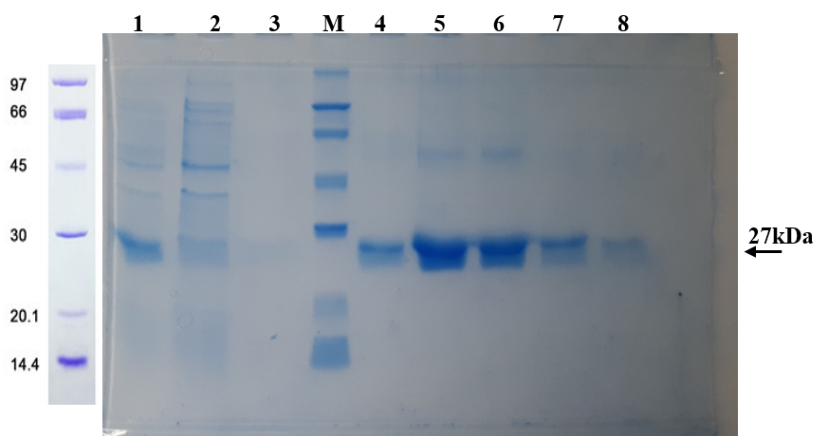


**Supplementary Figure S1c.** **ABD-GCSF protein.** The full size original image of Figure 2c: M: Protein Mw marker; #1, 2: Bacterial lysates before and after induction; #3, 4: Eluted proteins.


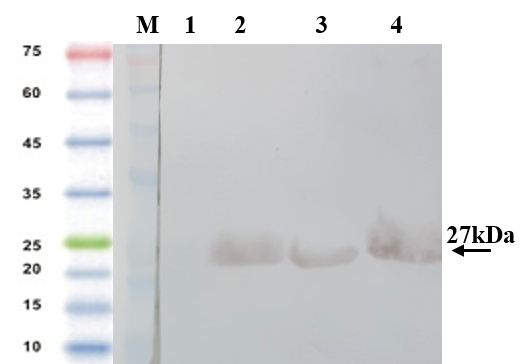


**Supplementary Figure 2**. Ellman’s assay plate.


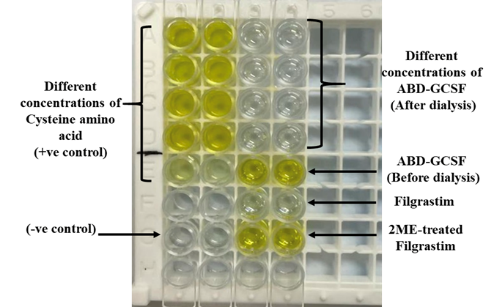


**Supplementary Figure 3.** 3D image of ABD-GCSF produced by PyMOL: a) The free cysteine residue buried within the molecule; b) Two disulfide bonds representing 4 cysteine residues which one of them is on the surface.

| a)  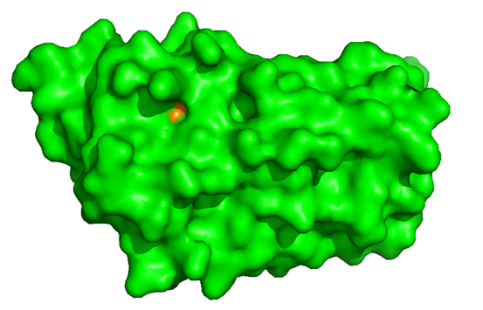 | b)  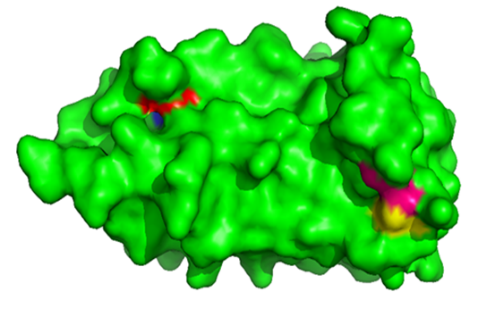 |
| --- | --- |

**Supplementary Figure 4.** Dynamic light scattering (DLS) for measurement of ABD-GCSF average diameter in double distilled water (pH 7.4) at 25°C.


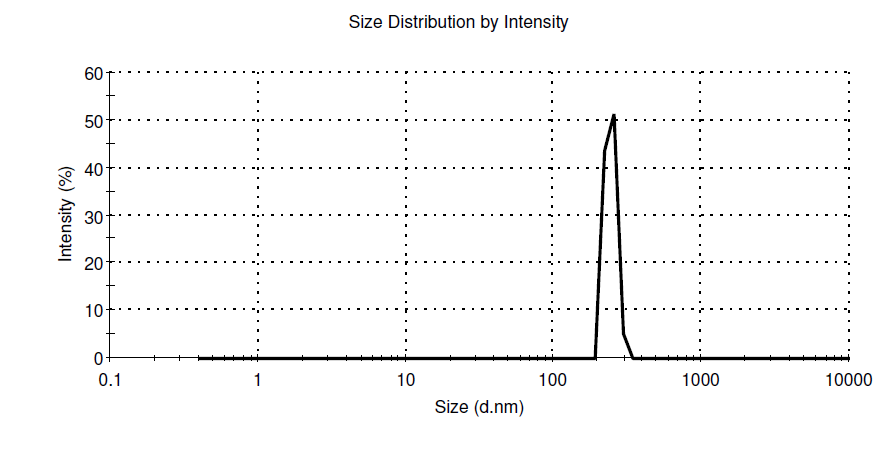


**Supplementary Figure 5.** Blood cell counts after administration of GCSF derivatives. **(a)** Filgrastim. **(b)** PEG-Filgrastim. **(c)** ABD-GCSF. Data are means±SE of 3 random rats/group.

| **a)**  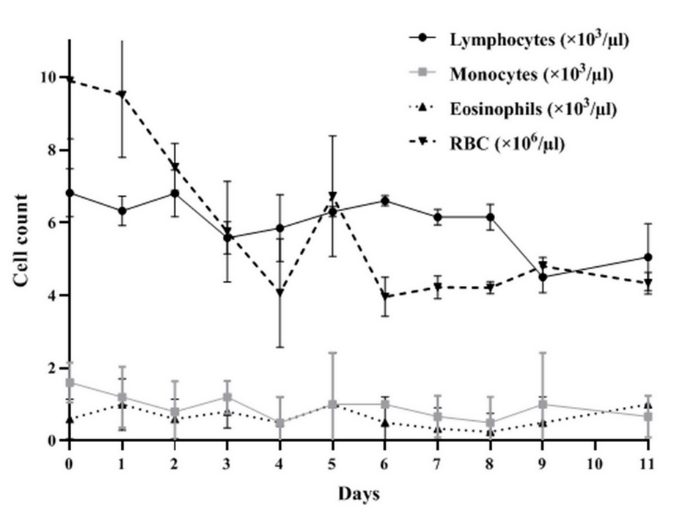 |
| --- |
| **b)**  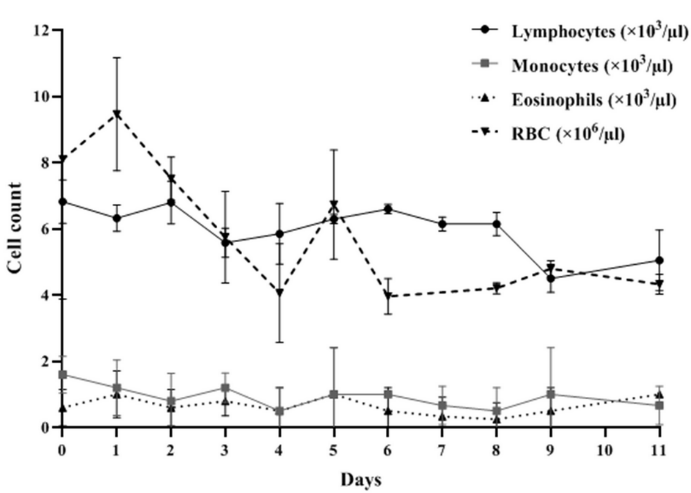 |
| **c)**  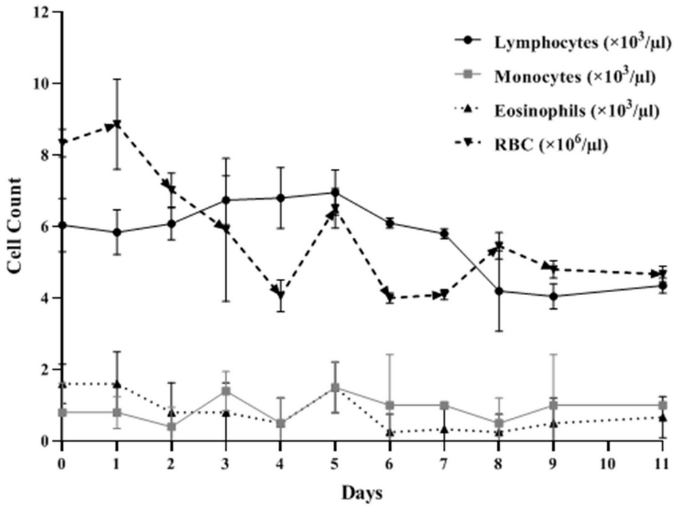 |

**Supplementary Figure 6.** SDS-PAGE quality assessment of the purified refolded ABD-GCSF protein: #1) Unfolded purified protein before dialysis; #2) Refolded protein after dialysis; #3) Concentrated protein using Centriprep-3kDa; M: Protein Mw marker.


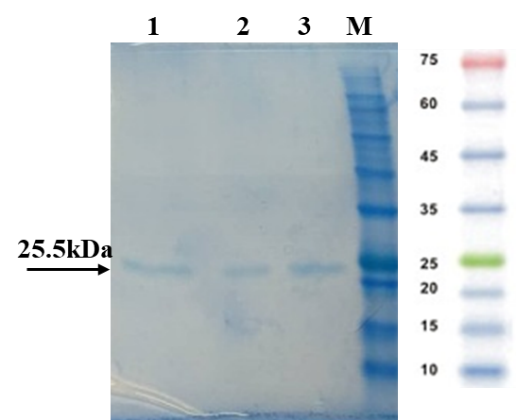


**Supplementary Figure** **7**. Nucleotide and amino acid sequences of ABD-GCSF expression cassette. Bolded, underlined and gray amino acids represent ABD094, GCSF and linker amino acids, respectively.

**
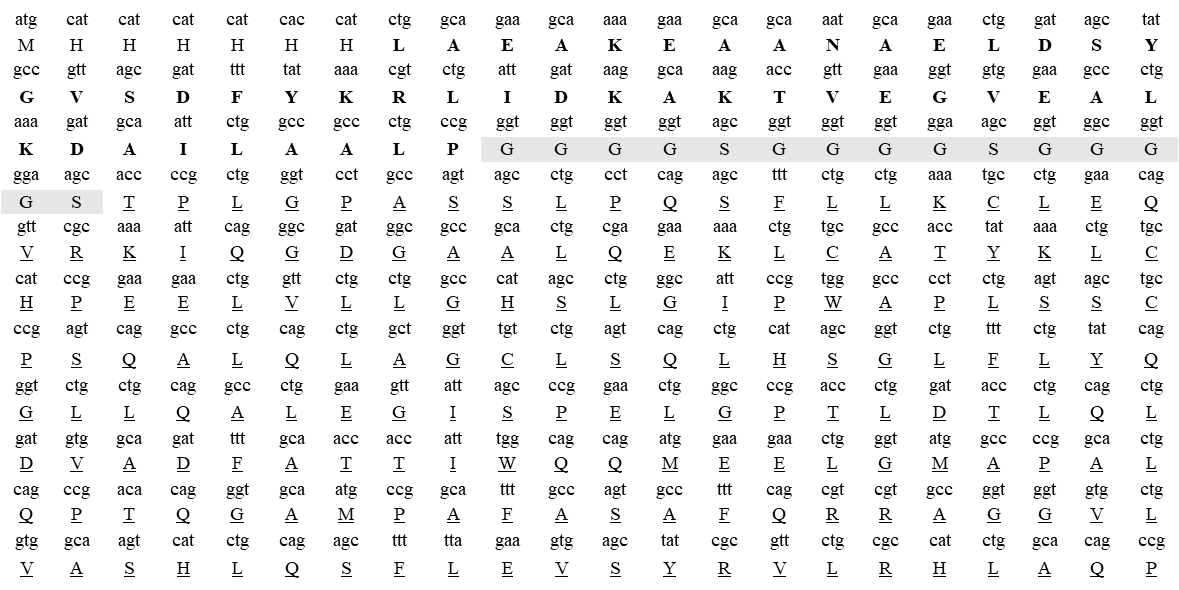
**
